# Epidemiological Patterns of Skin Disease in Saudi Arabia: A Systematic Review and Meta-Analysis

**DOI:** 10.1155/2020/5281957

**Published:** 2020-10-27

**Authors:** Mohammad Almohideb

**Affiliations:** King Saud Bin Abdulaziz University for Health Sciences, College of Medicine, Riyadh, Saudi Arabia

## Abstract

**Background:**

Large epidemiological studies on patterns of skin diseases in Saudi Arabia are scarce. Therefore, this systematic review and meta-analysis was conducted to gather available epidemiologic data describing the pattern of skin diseases in different geographical areas in Saudi Arabia.

**Methods:**

A comprehensive literature search of articles was conducted in PubMed, SCOPUS, and Web of Science through October 2019. We included all published cross-sectional studies that provided data on relevant incidence or prevalence of skin disease in Saudi Arabia. The risk of bias within the included cross-sectional studies was assessed using the Hoy tool for the prevalence studies. All statistical analysis was performed using the Comprehensive Meta-analysis software.

**Results:**

The present meta-analysis included 14 studies that reported the frequency of the skin disease patterns in different regions in Saudi Arabia with a total sample size of 30436 patients with an overall low risk of bias. The diseases of skin appendages and dermatitis were the most commonly reported skin diseases in Saudi Arabia (24.8% (95% CI, 24.3–25.3) and 24% (95% CI, 23.6%–24.6%), respectively). Skin infection represented about 18.5% (95% CI, 18.1%–19%), while the papulosquamous disorders represented 5.3% (95% CI, 5%–5.6%) of the skin diseases in Saudi Arabia. Skin cancers were pooled from only two studies. Basal cell carcinoma and squamous cell carcinoma were the most common malignant neoplasm in Saudi Arabia (51.4% and 22.5% of the malignant neoplasm, respectively), while malignant melanoma represents only 3.8% of the malignant skin cancer.

**Conclusion:**

Adnexal disorders and dermatitis are the most common skin disease in Saudi Arabia, followed by skin infection and pigmentary disorders. While skin cancer is more frequent than other countries, awareness campaigns should be initiated to increase knowledge about the harmful effect of long-term sun exposure.

## 1. Introduction

Skin, similar to other human organs, can be affected by all types of pathological changes, including hereditary, inflammatory, neoplastic, endocrinal, traumatic, and degenerative affection [1]. Epidemiological studies are important for understanding the implications of human disease. Identifying the incidence and prevalence of specific diseases is indispensable to decision making regarding the distribution of resources for clinical care and research [2]. For example, the management of skin disorders requires an appropriate diagnosis. Nondermatologists, as in the case of general practitioners, usually perform diagnoses and treatment of skin disorders in underserved areas, which highlights the importance of providing a comprehensive review of the skin disease patterns in each specific region; in addition to further focus on educating nondermatologists regarding some common skin conditions they might encounter [3–5].

The incidence and prevalence of skin disorders are mainly related to the ethnic and the genetic constitution of the community. In addition, hygiene, dietary style, social background, and weather conditions are important contributing factors.

Population-based epidemiological studies of skin disease are relatively inadequate. Because these epidemiologic studies have been published through many decades in different journals, many dermatologists are not aware of these data [2].

In Saudi Arabia, the large epidemiological studies on patterns of skin diseases are scanty. Previous epidemiological studies in Saudi Arabia investigated the pattern of skin and skin-related diseases in different regions of Saudi Arabia such as Madinah, Al-Khobar, Jeddah, Al-Baha, Hail, Abha, Qassim, and Najran [6–15], which reflect the pattern of skin and skin-related diseases in Saudi Arabia.

Therefore, this systematic review and meta-analysis was conducted to gather available epidemiological data describing the pattern of skin diseases and investigate the current evidence of frequency, type, and distribution of skin disorders in different geographical areas in Saudi Arabia.

## 2. Methods

All steps of this systematic review were performed in strict compliance with the Cochrane handbook of systematic reviews and meta-analysis [16] in addition to following the preferred reporting items for systematic reviews and meta-analyses (PRISMA statement guidelines) during the drafting process of this manuscript [17].

### 2.1. Literature Search Strategy

The following medical electronic databases were searched: PubMed, SCOPUS, and Web of Science through October 2019 using the following query: [(prevalence OR incidence OR epidemiology OR pattern of skin disease OR skin disease pattern)] AND (melanocytic nevi OR Vitiligo OR Melasma OR Dermatitis OR eczema OR acne OR alopecia OR fungal skin disease OR cutaneous leishmaniasis OR scabies OR warts OR chicken pox OR herpes simplex OR herpes zoster OR onychomycosis OR tinea OR dermatophytosis OR candidiasis OR pityriasis versicolorversicolor OR psoriasis OR Lichen planus OR pityriasis rosea OR skin cancer OR skin malignancy OR skin carcinoma OR melanoma) AND Saudi Arabia. The bibliography of eligible studies was searched to find relevant articles.

### 2.2. Eligibility Criteria and Study Selection

All published cross-sectional studies that provided data on relevant incidence or prevalence of skin disease in Saudi Arabia were included. Excluded articles included all studies with a small sample size (less than 100 patients), reviews, case reports, conference abstracts, or case series, studies with self-reported data unless diagnoses were validated by a trained physician, studies on specific ethnic or social groups, non-English articles, and duplicate references.

Eligibility screening was conducted in two steps, each by two independent reviewers (MA and MA) as follows: (a) title and abstract screening for matching the inclusion criteria and (b) full-text screening for eligibility to meta-analysis. Disagreements were resolved upon the opinion of a third reviewer (HA).

### 2.3. Data Extraction

Two independent reviewers (MA and MA) extracted the data that included the following: (a) general characteristics of each study including study setting, study design, sample size; (b) patients' baseline characteristics of each study including age, gender, and nationality; (c) types and proportion of the reported skin diseases; (d) risk of bias criteria.

### 2.4. Risk of Bias Assessment

To assess the risk of bias within the included cross-sectional studies, two independent reviewers (MA and MA) used the risk of bias assessment tool developed by Hoy et al. [18] for the prevalence studies. The domains of risk of bias assessment were presented in Supplementary file 1.

### 2.5. Data Synthesis

All statistical analysis was performed using the Comprehensive Meta-Analysis software (CMA version 3) for Windows. The mean proportions of skin diseases were pooled in a meta-analysis model, using the Mantel–Haenszel method. The analysis was performed under the fixed-effects model for homogeneous data and the random-effects model for heterogeneous data. Heterogeneity among studies was assessed using *I*^2^ test and -value from the chi-squared test of heterogeneity. Values of *I*^2^ > 50 are significant determinants of heterogeneity among studies [19].

## 3. Results

### 3.1. Results of the Literature Search

**Figure 1:**
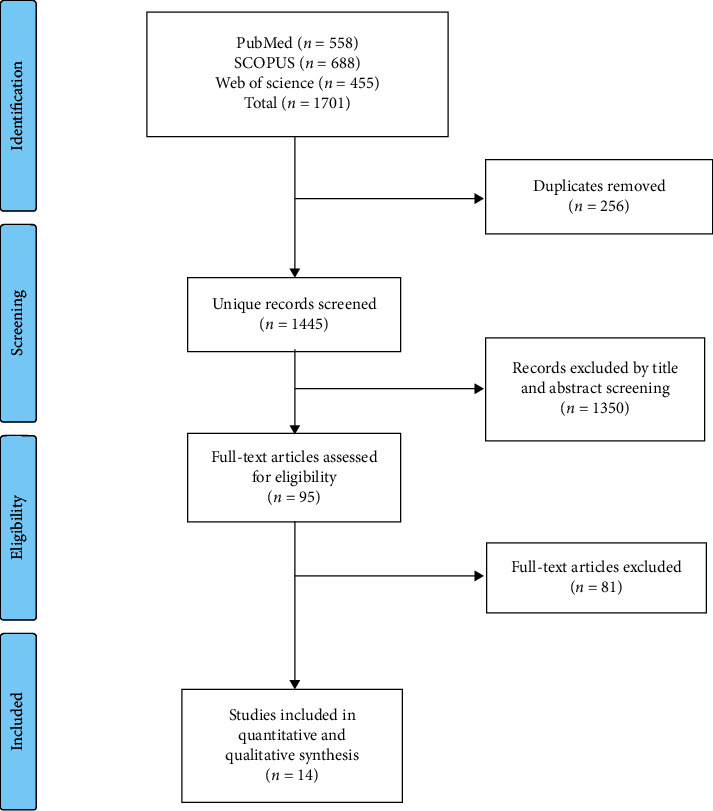
PRISMA flow diagram.

Our search yielded a total of 1701 studies. Following screening and excluding duplicates, we remained with 95 studies that entered full-text screening. Finally, 14 studies were included in this systematic review as reported in the PRISMA flow diagram (Figure 1).

### 3.2. Summary of the Included Studies

The fourteen Saudi studies reported the frequency of the skin disease patterns in different regions in Saudi Arabia with a total sample size of 30436 patients. Only two studies reported the pattern of skin cancers only in southeastern and western regions in Saudi Arabia, with a total sample size of 395 patients with skin cancer [6, 10]. Baseline characteristics and summary of the included studies are reported in Table 1.

Overall low risk of bias was observed in accordance with the Hoy et al. [18] assessment tool for the prevalence studies. Summary of risk of bias assessment is reported in Table 2.

### 3.3. Pattern of Skin Diseases in Saudi Arabia

**Figure 2:**
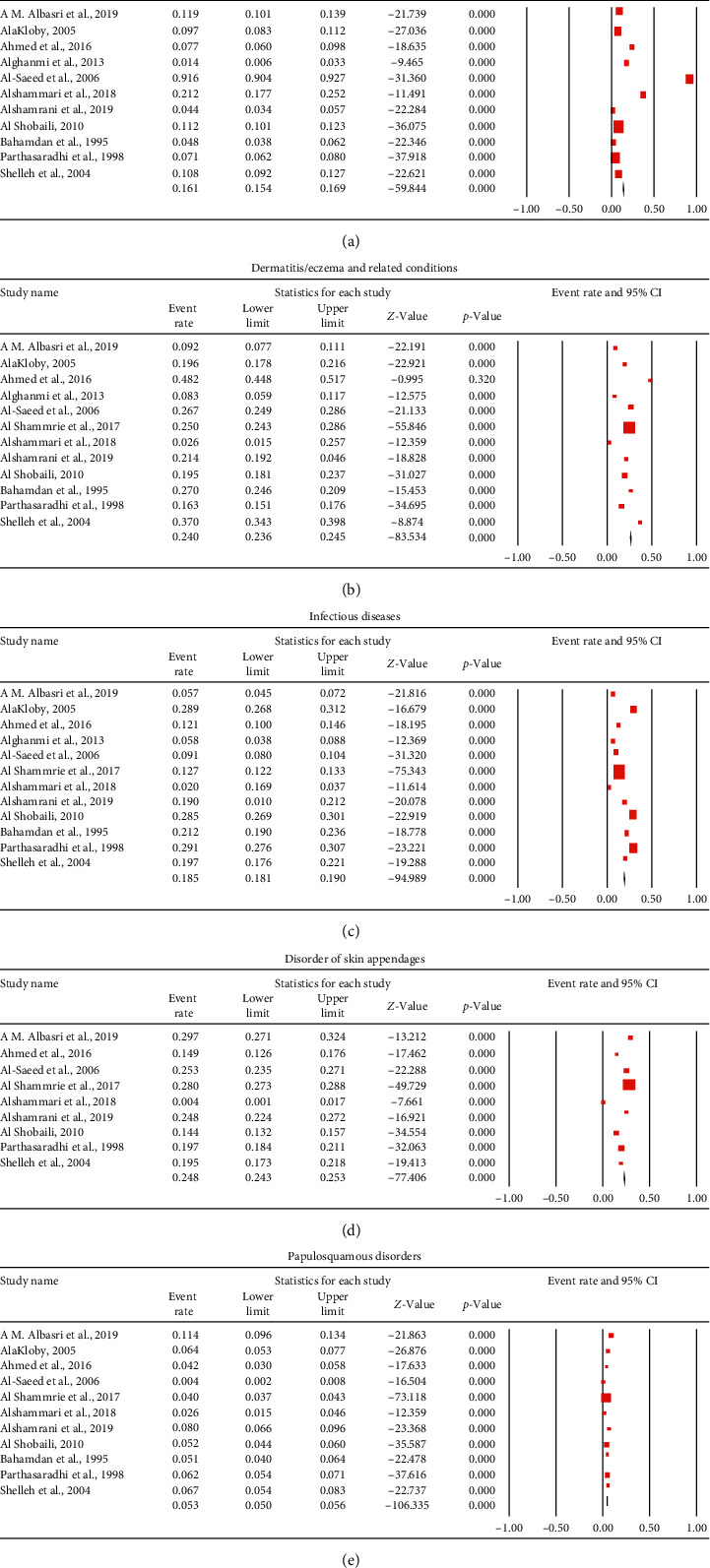
Forest plot of the pooled proportion for the prevalence of (a) pigmentary disorders, (b) dermatitis, (c) infectious diseases, (d) disorders of skin appendages, and (e) papulosquamous disorders.

The overall mean proportion of pigmentary disorders, as reported by 11 studies (16658 patients), was 16.1% (95% CI, 15.4%–16.9%) (Figure 2(a). The most common pigmentary disorder was the melanocytic nevi 54.2% (95% CI, 52.2%–56.1%) followed by postinflammatory pigmentation and vitiligo; 47% (95% CI, 45%–49%) and 6% (95% CI, 5.6%–6.3%), respectively Table 3 and Supplementary file 2. The pooled proportion of melanocytic nevi was significantly higher in males than females: OR 0.48 (95% CI, 0.33–0.69) (Table 4).

Considering dermatitis or eczema, the pooled proportion form 12 studies (29244 patients) resulted in an overall prevalence of 24% (95% CI, 23.6%–24.6%) (Figure 2(b)). Contact dermatitis and seborrheic dermatitis were common with a prevalence of 4.7% (95% CI 4.3%–5.1%) and 2.3% (95% CI, 2%–2.7%), respectively (Supplementary file 3). The pooled proportion of contact dermatitis was significantly higher in females than males: OR 0.82 (95% CI, 0.68–0.98) (Table 4).

Skin infection represented about 18.5% (95% CI, 18.1%–19%), as reported by 12 studies (29244 patients) (Figure 2(c)). Cutaneous leishmaniasis was the most common parasitic skin diseases 4% (95% CI, 3.7%–4.3%), while warts were the most common viral infection 7.2% (95% CI, 6.8%–7.5%) followed by chicken pox 6.9% (95% CI, 6.2%–7.7%). Bacterial skin diseases represented 3.3% (95% CI, 3%–3.6%). The most commonly reported fungal infections were dermatophytosis 6.6% (95% CI, 5.8%–7.4%) and onychomycosis 2.8% (95% CI, 2.3%–3.4%) (Supplementary file 4).

The pooled proportion of cutaneous leishmaniasis and warts was significantly higher in females than males: OR 2.07 (95% CI, 1.58–2.72) and OR 1.49 (95%CI, 1.25–1.77), while the bacterial infection was more common in males: OR 1.80 (95% CI, 1.41–2.30) (Table 4).

Regarding the diseases of skin appendages, the mean proportion was 24.8% (95% CI, 24.3–25.3) (Figure 2(d)) as reported by 9 studies (27177 patients). Alopecia and acne served a proportion of 7.5% (95% CI, 7.1%–7.8%) and 1.8% (95% CI, 1.7%–1.8%) (Supplementary file 5). The pooled proportion of acne was significantly higher in females than males: OR 0.57 (95% CI, 0.49–0.65) (Table 4).

On the other hand, papulosquamous disorders represented 5.3% (95% CI, 5%–5.6%) of the skin diseases in Saudi Arabia (Figure 2(e)), as reported by 11 studies (30076 patients). Psoriasis represents 3.9% (95% CI, 3.6%–4.1%), while Lichen planus represents 1.8% (95% CI, 1%–1.5%) (Supplementary file 6). The summary of the pooled proportion of the skin diseases pattern in Saudi Arabia is presented in Table 2.

**Figure 3:**
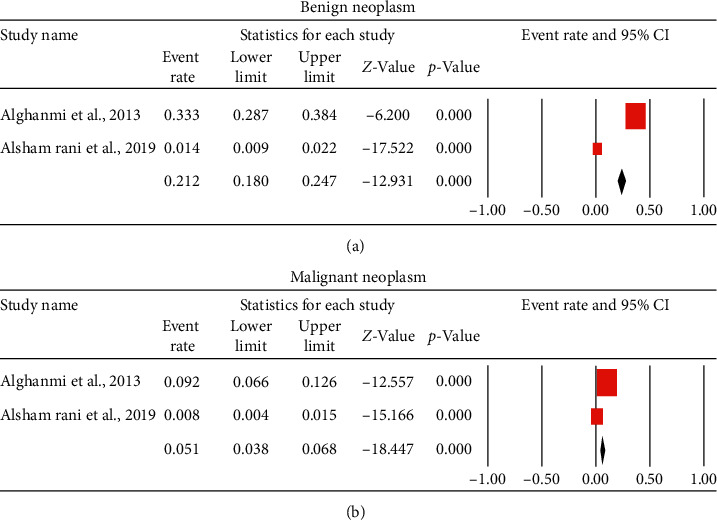
Forest plot of the pooled proportion for the prevalence of skin cancer: (a) benign neoplasm and (b) malignant neoplasm.

**Figure 4:**
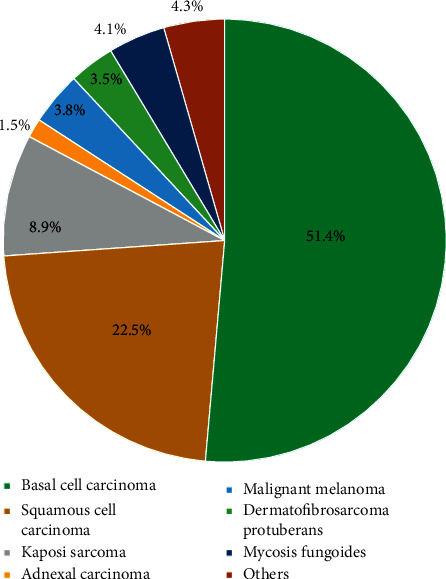
Pie chart summary of the pooled proportion for the prevalence malignant neoplasm.

Finally, the overall mean proportion of benign skin neoplasms, as reported by two studies (1244 patients) was 21.2% (95% CI, 18%–24.7%) (Figure 3(a)), while malignant neoplasms were 5%. Two studies reported the histological classification of skin cancer in Saudi Arabia. The pooled proportion from the two studies is reported in Figure 4. Basal cell carcinoma and squamous cell carcinoma were the most common malignant neoplasm in Saudi Arabia (51.4% and 22.5%, respectively). Malignant melanoma represents 3.8% of malignant skin cancers in Saudi Arabia.

## 4. Discussion

This systematic review presented a summary of population-based data describing the pattern of skin disease in Saudi Arabia. Previous studies investigated the pattern of skin and skin-related diseases in different regions of Saudi Arabia such as Madinah, Al-Khobar, Jeddah, Al-Baha, Hail, Abha, Qassim, and Najran through population-based cross-sectional studies, which reflect the true pattern of skin diseases in Saudi Arabia [6–15]. Although these included data did not cover every part of Saudi Arabia, the results are generalizable to other populations and reflect the current evidence of the frequency, type, and distribution of skin diseases in different geographical areas in Saudi Arabia.

The different patterns of skin disorders in different cities in Saudi Arabia might be justified by overcrowding and/or poor living conditions in some cities. Other ecological and environmental considerations should be considered.

Fourteen Saudi studies with a total sample size of 30436 were included in this systematic review and meta-analysis reporting the frequency of the skin disease patterns in different regions in Saudi Arabia. The average sample size of the individual studies is 2000 patients, which is small to determine the true pattern of skin disease in a large country as Saudi Arabia.

In the present study, dermatitis or eczema represented a high prevalence of skin disorders in Saudi Arabia, with an overall prevalence of 24% from a total sample size of 29244 patients. Contact dermatitis and seborrheic dermatitis were common. This prevalence is supported by various studies conducted in Saudi Arabia with reported prevalences of 19.6% [8], 48.2% [20], 37% [12], 21% [21], 19.5% [14], 25.7% [15], 16.31% [22], and 37% [23]. The high prevalence of dermatitis may contribute to climatical variability in different parts of Saudi Arabia.

Skin infections and infestations prevalence have been reported with an overall pooled proportion of 18.5% derived from 12 studies with an overall sample size of 29244 patients.

Cutaneous leishmaniasis was the most common parasitic skin diseases, while the warts were the most common viral infection followed by chickenpox. In addition, bacterial skin diseases represented 3.3% of the total prevalence of skin disease in Saudi Arabia.

Similar to the results of this study, a review of population-based studies from the Rochester epidemiology project showed that skin-related infections and infestations have a high prevalence rate. The incidence of herpes zoster has repeatedly been found to be very high (590 Per 100,000 person-years). The incidence of lower extremity cellulitis is also very common (213 Per 100,000 person-years) [2].

A recent study reported that 10.9% of the population had a fungal infection in Jeddah, Saudi Arabia [21], which is almost equal to that in Al-Khobar (9.6%) [21]. This high prevalence of fungal infection is probably attributed to the hot and humid climate in both coastal cities creating good media for fungal infections. Concomitantly, infections were the most common skin disease in Cairo, Egypt (15.83%) [24], and this finding was similarly reported in other developing countries where poor hygiene, low educational level, and poverty play important roles [25–28]. Interestingly, similar data showed a high prevalence of fungal infection in high socioeconomic countries in the same region such as Abu Dhabi (8.5%) and Qatar (11.4%) [21]. The present study reported that the most common fungal infections were dermatophytosis and onychomycosis.

Regarding skin appendages disorders, the mean prevalence was 24.8% in Saudi Arabia. Acne served the highest proportion of them. Saudi studies reported that acne is the second most common skin disease in Jeddah, Saudi Arabia [21]. This result was also reported in Al-Khobar [8], Najran [23], Hail [12], and Al-Qunfudah [20], while acne was the third most common in Asir [15]. In Cairo, Egypt, acne represented the second most common skin disease (6.11%), after scabies (9.26%), contact dermatitis (7.92%), and pityriasis versicolor (7.7%) [24]. Diseases of skin appendages in Iran [25] and Tunisia [28] represented the second most common skin disease with significant female predominance over men among all diseases of skin appendages.

This meta-analysis reported a 5.3% prevalence of papulosquamous disorders in an overall sample size of 30076 patients from 11 studies. Psoriasis represents 3.9%, while Lichen planus represents 1.8%. Papulosquamous disorders had a higher prevalence in Jaddah (8%), Al-jouf (7.4%), Najran (6.7), and Al-Khobar (6.5%). The prevalence was lower prevalence in Asir (4.8%) and Qunfudah (4.2%) [21].

In Cairo, Egypt, the most common skin disease was infections (45.4%) followed by hypersensitivity diseases (22.08%) and adnexal disorders (16.17%). Papulosquamous disorders represented about 4.69% of the total prevalence of skin diseases. Lastly, pigmentary disorders represented only 4.24% and skin neoplasm 1.29%.

In this meta-analysis of Saudi studies, the overall mean proportion of pigmentary disorders as reported by 11 studies was 16.1% from a total sample size of 16658 patients. Melanocytic nevi and vitiligo were the most common pigmentary disorders in Saudi Arabia.

Lastly, the overall prevalence of benign skin neoplasms as reported by two studies was 21.2%, while malignant neoplasms were 5%. Basal cell carcinoma and squamous cell carcinoma were the most common malignant cancer in Saudi Arabia. Malignant melanoma represents 3.8% of malignant skin cancer. These figures are believed to be not representable as skin cancer is barely seen in Saudi Arabia.

The many strength points of this meta-analysis are the large numbers of included studies with a large sample size that covers most of the Saudi regions. Besides, we reported the first class of evidence form studies with low risk of bias according to the Hoy et al. [18] assessment tool for the prevalence studies.

For future researches, we recommend a comprehensive nationwide population-based study to identify the prevalence of skin diseases in Saudi Arabia.

In conclusion, Adnexal disorders and dermatitis are the most common skin disease in Saudi Arabia followed by skin infection and pigmentary disorders. Dermatological educational programs for primary healthcare physicians should be implemented to provide rapid detection of cases at early stagesbib19.

## Figures and Tables

**Table 1:** Baseline characteristics and summary of the included studies.

Study ID	Region	Type of study	Sample size	Nationality		Time Period	Criteria of classification	Age (years)	Gender		Most common age
				Saudi	Expatriates				Male, *N* (%)	Female, *N* (%)	
Albasri et al. 2018	Madinah	Retrospective hospital-based study (pathology based)	202	NR		Jan 2006 to Dec 2017	NR	60.1 ± 15	139 (68.8%)	63 (31.2%)	60–69
Albasri et al. 2019	Madinah	Retrospective hospital-based study (pathology based)	1125	NR		Jan 2006 to Dec 2017	ICD-10	36.9 ± 9.8	579 (51.5%)	546 (48.5%)	20–49
Alakloby 2005	King Fahd Hospital, Al-Khobar, eastern province	Prospective hospital-based study	1676	432 (85.5%)	244 (14.6%)	Aug 2002 to Jul 2003	ICD	NR	820 (49%)	856 (51%)	Adults 1,274 (76%) 13 years, and children 402 (24%) <13 years
Ahmed et al. 2016	Qunfudah region	Retrospective hospital-based study	792	NR		Oct 2015 to Sep 2016	NR	Mean age in females (29.2) and males (31.1)	342 (43.2%)	450 (56.8%)	15–24 (25.1%)
Alghanmi et al. 2013	Jeddah	Retrospective hospital-based study (pathology based)	360		NR	Jan 2005 to Dec 2010	NR	NR	F > M	F > M	>46
Al-Maghrabi et al. 2004	Al-Baha, south western region	Retrospective hospital-based study (pathology based)	193	189 (98%)	4 (2%)	Jan 1990 to Jul 2003	NR	Mean age 62.2 years	119 (62%)	74 (38%)	60–80
Al-Saeed et al. 2006	Al-Khobar city, eastern province	Cross-sectional study	2239	1844 (82.4%)	395 (17.6%)	Jan to Mar 2003	ICD-10	10.49 ± 2.64	Female schoolchildren		6–17
Al Shammrie et al. 2017	Hail region	Retrospective hospital-based study	13778	12,574 (92%)	1204 (8%)	Jan 2008 to Dec 2014 with missing two years (2010–2011)	NR	NR	5473 (40%)	8305 (60%)	NR
Alshammari et al. 2018	Hail region	Retrospective hospital-based study (histopathology based)	458	NR	NR	Jan 2014 to Apr 2017	NR	34.29 ± 17.5	242 (52.8%)	216 (47.2%)	30–39
Alshamrani et al. 2019	Jeddah, western region	Retrospective hospital-based study	1244	809 (65%)	435 (35%)	January 2017 to December 2017	ICD-10	35 ± 3.8	365 (29.3%)	879 (70.7%	Children 127 (10.2%) (<13 years), adults 982 (78.9%) (13–60 years), and elders 135 (10.9%) (>60 years)
Al Shobaili 2010	Qassim region	Prospective hospital-based study	3051	3051 (100%)	0	Mar 2008 to Feb 2009	NR	25.3 ± 14.9	1786 (58.5%)	1265 (41.5%)	5–34
Bahamdan et al. 1995	Abha, southern region, Saudi Arabia	Prospective hospital-based study	1223	939 (76.8%)	284 (23.2%)	Mar 1992 to Mar 1993	ICD-9	NR	667 (54.54%)	556 (45.46%)	Adults 848 (>13 years) and children 375 (30.7%) (<13 years).
Parthasaradhi et al. 1998	Hail region, Saudi Arabia	Prospective hospital-based study	3298	2772 (84.05%)	526 (15.95%)	Jul 1995 to Jun 1997	ICD-9	NR	1819 (55.16%)	1479 (44.84%)	Adults 2535 (>13 years) and children 763 (<13 years)
Shelleh et al. 2004	Najran	Retrospective hospital-based study (pathology based)	1192	1003 (84.1%)	189 (15.9%)	Dec 2000 to Dec 2001	ICD-9	NR	562 (47.1%)	630 (52.9%)	Children 302 (25.3%) and adults 890

**Table 2:** Evaluation of the risk of bias in included primary studies.

Study ID	Q1	Q2	Q3	Q4	Q5	Q6	Q7	Q8	Q9	Q10	Overall risk of study bias
Albasri et al. 2018	Low risk	Low risk	Low risk	Low risk	Low risk	Low risk	Low risk	Low risk	Low risk	Low risk	Low risk
Albasri et al. 2019	High risk	Low risk	Low risk	Low risk	Low risk	Low risk	Low risk	Low risk	Low risk	Low risk	Low risk
Alakloby 2005	High risk	Low risk	Low risk	Low risk	Low risk	Low risk	Low risk	Low risk	Low risk	Low risk	Low risk
Ahmed et al. 2016	High risk	High risk	High risk	Low risk	Low risk	Low risk	High risk	Low risk	Low risk	Low risk	Moderate risk
Alghanmi et al. 2013	Low risk	Low risk	Low risk	Low risk	Low risk	Low risk	Low risk	Low risk	Low risk	Low risk	Low risk
Al-Maghrabi et al. 2004	Low risk	Low risk	Low risk	Low risk	Low risk	Low risk	Low risk	Low risk	Low risk	Low risk	Low risk
Al-Saeed et al. 2006	High risk	Low risk	Low risk	Low risk	Low risk	Low risk	Low risk	Low risk	High risk	Low risk	Low risk
Al Shammrie et al. 2017	Low risk	Low risk	Low risk	Low risk	Low risk	Low risk	Low risk	Low risk	High risk	Low risk	Low risk
Alshammari et al. 2018	High risk	Low risk	Low risk	Low risk	Low risk	Low risk	Low risk	Low risk	Low risk	Low risk	Low risk
Alshamrani et al. 2019	High risk	Low risk	Low risk	Low risk	High risk	Low risk	Low risk	Low risk	High risk	Low risk	Low risk
Al Shobaili 2010	Low risk	Low risk	Low risk	Low risk	Low risk	Low risk	Low risk	Low risk	Low risk	Low risk	Low risk
Bahamdan et al. 1995	Low risk	Low risk	Low risk	Low risk	Low risk	Low risk	Low risk	Low risk	Low risk	Low risk	Low risk
Parthasaradhi et al. 1998	Low risk	Low risk	Low risk	Low risk	Low risk	Low risk	Low risk	Low risk	Low risk	Low risk	Low risk
Shelleh et al. 2004	Low risk	Low risk	Low risk	Low risk	Low risk	Low risk	Low risk	Low risk	Low risk	Low risk	Low risk

List of the 10 questions (Q1–Q10) applied to the studies: Q1. Was the study's target population a close representation of the national population in relation to relevant variables, e.g., age, sex, occupation? Q2. Was the sampling frame a true or close representation of the target population? Q3. Was some form of random selection used to select the sample, OR, was a census undertaken? Q4. Was the likelihood of nonresponse bias minimal? Q5. Were data collected directly from the subjects (as opposed to a proxy)? Q6. Was an acceptable case definition used in the study? Q7. Was the study instrument that measured the parameter of interest shown to have reliability and validity (if necessary)? Q8. Was the same mode of data collection used for all subjects? Q9. Was the length of the shortest prevalence period for the parameter of interest appropriate? Q10. Were the numerator(s) and denominator(s) for the parameter of interest appropriate?

**Table 3:** Pooled proportion of the skin diseases pattern in Saudi Arabia.

Skin diseases	Pooled proportion (%)	95% CI
Pigmentary Disorder		
Melanocytic nevi	54.2	52.2%–56.1%
Postinflammatory hypo- and hyperpigmentation	47	45%–49%
Vitiligo	6	5.6%–6.3%
Melasma	2.5	2.2%–3%

Dermatitis/eczema and related conditions		
Contact dermatitis	4.7	4.3%–5.1%
Seborrheic dermatitis	2.3	2%–2.7%
Atopic dermatitis	1.2	1.1%–1.3%
Pityriasis alba	1.6	1.2%–2.1%

Disorder of skin appendages		
Acne	1.8	1.7%–1.8%
Alopecia	7.5	7.1%–7.8%

Infectious diseases		
Cutaneous leishmaniasis	4	3.7%–4.3%
Scabies	0.6	0.4%–0.8%
Warts	7.2	6.8%–7.5%
Chicken pox	6.9	6.2%–7.7%
Herpes simplex	1.2	0.9%–1.5%
Herpes zoster	1.8	1.5%–2.2%
Bacterial	3.3	3%–3.6%
Onychomycosis	2.8	2.3%–3.4%
Tinea	2.3	2.1%–2.5%
Dermatophytosis	6.6	5.8%–7.4%
Candidiasis	1.6	1.3%–2%
Pityriasis versicolor	1.2	0.8%–1.7%

Papulosquamous disorders		
Psoriasis	3.9	3.6%–4.1%
Lichen planus	1.8	1.6%–2.1%
Pityriasis rosea	1.2	1%–1.5%

**Table 4:** Comparison between males and females regarding the skin diseases pattern in Saudi Arabia.

Skin diseases	Males	Total	Females	Total	Odds ratio (95% CI)
Pigmentary Disorder					
Melanocytic nevi	48	944	92	1425	0.48 (0.33, 0.69)
Postinflammatory hypo and hyperpigmentation	20	1185	41	1735	0.58 (0.33, 1.00)
Vitiligo	149	3671	119	3770	1.20 (0.94, 1.55)
Melasma	18	3218	86	2881	0.18 (0.11, 0.31)

Dermatitis/eczema and related conditions					
Contact dermatitis	222	4812	270	4946	0.82 (0.68, 0.98)
Seborrheic dermatitis	93	4250	88	4316	1.13 (0.84, 1.52)
Atopic dermatitis	501	4812	487	4946	1.11 (0.97, 1.27)

Disorder of skin appendages					
Acne	353	3566	636	3844	0.57 (0.49, 0.65)
Alopecia	179	4145	378	4390	0.48 (0.40, 0.58)

Infectious diseases					
Cutaneous leishmaniasis	185	4250	78	4316	2.07 (1.58, 2.72)
Scabies	25	3671	21	3770	1.19 (0.67, 2.12)
Warts	356	4250	239	4316	1.49 (1.25, 1.77)
Chicken pox	46	3306	25	2891	1.53 (0.94, 2.50)
Herpes simplex	28	3671	21	3770	1.44 (0.80, 2.58)
Herpes zoster	72	3430	62	3460	1.23 (0.86, 1.75)
Bacterial	190	4549	118	4169	1.80 (1.41, 2.30)
Dermatophytosis	192	3430	157	3460	1.53 (1.22, 1.91)
Candidiasis	41	3671	53	3770	0.69 (0.46, 1.05)
Pityriasis versicolor	49	1852	28	2291	2.22 (1.37, 3.58)

Papulosquamous disorders					
Psoriasis	163	4250	159	4316	1.11 (0.88, 1.39)
Lichen planus	63	4250	84	4316	0.78 (0.56, 1.09)
Pityriasis rosea	46	4250	55	4316	0.83 (0.56, 1.22)

## Supplementary Materials

Supplementary Materials Supplementary file 1: risk of bias assessment tool. Supplementary file 2: forest plot of the pooled proportion for the prevalence of pigmentary disorders. Supplementary file 3: forest plot of the pooled proportion for the prevalence of dermatitis/eczema and related conditions. Supplementary file 4: forest plot of the pooled proportion for the prevalence of skin infections. Supplementary file 5: forest plot of the pooled proportion for the prevalence of diseases of skin appendages. Supplementary file 6: forest plot of the pooled proportion for the prevalence of papulosquamous disorders.
