# Supplementary material for: Epidemiological Patterns of Skin Disease in Saudi Arabia: A Systematic Review and Meta-Analysis
Source: Dermatol Res Pract. 2020 Oct 27;2020:5281957. doi: 10.1155/2020/5281957 (PMC7641721; doi:10.1155/2020/5281957)
Supplement: Supplementary Materials — Supplementary file 1: risk of bias assessment tool. Supplementary file 2: forest plot of the pooled proportion for the prevalence of pigmentary disorders. Supplementary file 3: forest plot of the pooled proportion for the prevalence of dermatitis/eczema and related conditions. Supplementary file 4: forest plot of the pooled proportion for the prevalence of skin infections. Supplementary file 5: forest plot of the pooled proportion for the prevalence of diseases of skin appendages. Supplementary file 6: forest plot of the pooled proportion for the prevalence of papulosquamous disorders. [file 5281957.f1.zip › 5281957/Supplementary file 1 Risk of bias tool used (1).pdf]

Supplementary file 1: Risk of Bias Tool

Risk of bias item	Criteria for answers (please circle one option)	Additional notes and examples
External Validity		
1. Was the study's target population **a close representation** of the national population in relation to relevant variables, e.g. age, sex, occupation?	• **Yes (LOW RISK):** The study's target population was a close representation of the national population.<br>• **No (HIGH RISK)**: The study's target population was clearly NOT representative of the national population.	The **target population** refers to the group of people or entities to which the results of the study will be generalised. Examples:<br>• The study was a national health survey of people 15 years and over and the sample was drawn from a list that included all individuals in the population aged 15 years and over. The answer is: **Yes (LOW RISK).**<br>• The study was conducted in one province only, and it is not clear if this was representative of the national population. The answer is: **No (HIGH RISK).**<br>• The study was undertaken in one village only and it is clear this was not representative of the national population. The answer is: **No (HIGH RISK).**
2. Was the sampling frame a **true or close representation** of the target population?	• **Yes (LOW RISK):** The sampling frame was a **true or close** representation of the target population.<br>• **No (HIGH RISK)**: The sampling frame was NOT a **true or close** representation of the target population.	The **sampling frame** is a list of the sampling units in the target population and the study sample is drawn from this list. Examples:<br>• The sampling frame was a list of almost every individual within the target population. The answer is: **Yes (LOW RISK)**.<br>• The cluster sampling method was used and the sample of clusters/villages was drawn from a list of all villages in the target population. The answer is: **Yes (LOW RISK)**.<br>• The sampling frame was a list of just one particular ethnic group within the overall target population, which comprised many groups. The answer is: **No (HIGH RISK).**
3. Was some form of **random selection** used to select the sample, OR, was a census undertaken?	• **Yes (LOW RISK):** A census was undertaken, OR, some form of random selection was used to select the sample (e.g. simple random sampling, stratified random sampling, cluster sampling, systematic sampling).<br>• **No (HIGH RISK)**: A census was NOT undertaken, AND some form of random selection was NOT used to select the sample.	A census collects information from every unit in the sampling frame. In a survey, only part of the sampling frame is sampled. In these instances, random selection of the sample helps minimise study bias. Examples:<br>• The sample was selected using simple random sampling. The answer is: **Yes (LOW RISK)**.<br>• The target population was the village and every person in the village was sampled. The answer is: **Yes (LOW RISK)**.<br>• The nearest villages to the capital city were selected in order to save on the cost of fuel. The answer is: **No (HIGH RISK).**
4. Was the likelihood of **non-response bias minimal?**	• **Yes (LOW RISK):** The response rate for the study was >/=75%, OR, an analysis was performed that showed no significant difference in relevant demographic characteristics between responders and non-responders<br>• **No (HIGH RISK)**: The response rate was <75%, and if any analysis comparing responders and non-responders was done, it showed a significant difference in relevant demographic characteristics between responders and non-responders.	Examples:<br>• The response rate was 68%; however, the researchers did an analysis and found no significant difference between responders and non-responders in terms of age, sex, occupation and socio-economic status. The answer is: **Yes (LOW RISK)**.<br>• The response rate was 65% and the researchers did NOT carry out an analysis to compare relevant demographic characteristics between responders and non-responders. The answer is: **No (HIGH RISK)**.<br>• The response rate was 69% and the researchers did an analysis and found a significant difference in age, sex and socio-economic status between responders and non-responders. The answer is: **No (HIGH RISK)**.

Internal Validity		
5. Were data collected **<u>directly from the subjects</u>** (as opposed to a proxy)?	• **Yes (LOW RISK):** All data were collected directly from the subjects.<br>• **No (HIGH RISK)**: In some instances, data were collected from a proxy.	A proxy is a representative of the subject. Examples:<br>• All eligible subjects in the household were interviewed separately. The answer is: **Yes (LOW RISK)**.<br>• A representative of the household was interviewed and questioned about the presence of low back pain in each household member. The answer is: **No (HIGH RISK)**.
6. Was an acceptable case definition used in the study?	• **Yes (LOW RISK):** An acceptable case definition was used.<br>• **No (HIGH RISK)**: An acceptable case definition was <u>NOT</u> used.	• For a study on low back pain, the following case definition was used: "Low back pain is defined as activity-limiting pain lasting more than one day in the area on the posterior aspect of the body from the bottom of the 12th rib to the lower gluteal folds." The answer is: **Yes (LOW RISK)**.<br>• For a study on back pain, there was no description of the specific anatomical location 'back' referred to. The answer is: **No (HIGH RISK)**.<br>• For a study on osteoarthritis, the following case definition was used: "Symptomatic osteoarthritis of the hip or knee, radiologically confirmed as Kellgren-Lawrence grade 2-4". The answer is: LOW RISK.
7. Was the study instrument that measured the parameter of interest (e.g. prevalence of low back pain) shown to have **<u>reliability and validity (if necessary)</u>**?	• **Yes (LOW RISK):** The study instrument had been shown to have reliability and validity (if this was necessary), e.g. test-re-test, piloting, validation in a previous study, etc.<br>• **No (HIGH RISK)**: The study instrument had <u>NOT</u> been shown to have reliability or validity (if this was necessary).	• The authors used the COPCORD questionnaire, which had previously been validated. They also tested the inter-rater reliability of the questionnaire. The answer is: **Yes (LOW RISK)**.<br>• The authors developed their own questionnaire and did not test this for validity or reliability. The answer is: **No (HIGH RISK)**.
8. Was the **<u>same mode of data collection</u>** used for all subjects?	• **Yes (LOW RISK):** The same mode of data collection was used for all subjects.<br>• **No (HIGH RISK)**: The same mode of data collection was NOT used for all subjects.	The mode of data collection is the method used for collecting information from the subjects. The most common modes are face-to-face interviews, telephone interviews and self-administered questionnaires. Examples:<br>• All eligible subjects had a face-to-face interview. The answer is: **Yes (LOW RISK)**.<br>• Some subjects were interviewed over the telephone and some filled in postal questionnaires. The answer is: **No (HIGH RISK)**.
9. Was the **<u>length of the shortest prevalence period</u>** for the parameter of interest appropriate?	• **Yes (LOW RISK):** The shortest prevalence period for the parameter of interest was appropriate (e.g. point prevalence, one-week prevalence, one-year prevalence).<br>• **No (HIGH RISK)**: The shortest prevalence period for the parameter of interest was not appropriate (e.g. lifetime prevalence)	The prevalence period is the period that the subject is asked about e.g. "Have you experienced low back pain over the previous year?" In this example, the prevalence period is one year. The longer the prevalence period, the greater the likelihood of the subject forgetting if they experienced the symptom of interest (e.g. low back pain). Examples:<br>• Subjects were asked about pain over the past week. The answer is: **Yes (LOW RISK)**.<br>• Subjects were only asked about pain over the past three years. The answer is: **No (HIGH RISK)**.
10. Were the **<u>numerator(s) and denominator(s)</u>** for the parameter of interest appropriate?	• **Yes (LOW RISK):** The paper presented appropriate numerator(s) AND denominator(s) for the parameter of interest (e.g. the prevalence of low back pain).<br>• **No (HIGH RISK)**: The paper did present numerator(s) AND denominator(s) for the parameter of interest but one or more of these were inappropriate.	There may be errors in the calculation and/or reporting of the numerator and/or denominator. Examples:<br>• There were no errors in the reporting of the numerator(s) AND denominator(s) for the prevalence of low back pain. The answer is: **Yes (LOW RISK)**.<br>• In reporting the overall prevalence of low back pain (in both men and women), the authors accidentally used the population of women as the denominator rather than the combined population. The answer is: **No (HIGH RISK).**
11. Summary item on the overall risk of study bias		
• **LOW RISK OF BIAS:** Further research is <u>very unlikely</u> to change our confidence in the estimate.<br>• **MODERATE RISK OF BIAS:** Further research is <u>likely</u> to have an important impact on our confidence in the estimate and may change the estimate.		

- **HIGH RISK OF BIAS:** Further research is very likely to have an important impact on our confidence in the estimate and is likely to change the estimate.
