# Supplementary material for: Epidemiological Patterns of Skin Disease in Saudi Arabia: A Systematic Review and Meta-Analysis
Source: Dermatol Res Pract. 2020 Oct 27;2020:5281957. doi: 10.1155/2020/5281957 (PMC7641721; doi:10.1155/2020/5281957)

Supplementary File 2

Melanocytic nevi

Study name	Event rate	Lower limit	Upper limit	Z-Value	p-Value
A. M. Albasri et al., 2019	0.118	0.101	0.138	-21.760	0.000
Al-Saeed et al., 2006	0.684	0.664	0.703	16.969	0.000
Alshamrani et al., 2019	0.006	0.003	0.012	-13.652	0.000
	0.542	0.522	0.561	4.123	0.000

Event rate and 95% CI

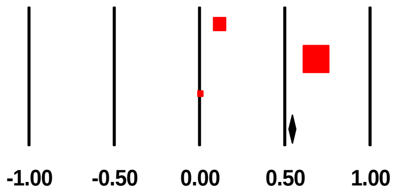

Post inflammatory hypo and hyper pigmentation

Study name	Event rate	Lower limit	Upper limit	Z-Value	p-Value
Alakloby, 2005	0.030	0.023	0.039	-24.250	0.000
Al-Saeed et al., 2006	0.566	0.546	0.587	6.258	0.000
Alshamrani et al., 2019	0.009	0.005	0.016	-15.583	0.000
	0.470	0.450	0.490	-2.982	0.003

Event rate and 95% CI

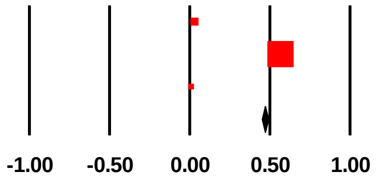

Vitiligo

Study name	Event rate	Lower limit	Upper limit	Z-Value	p-Value
Alakloby, 2005	0.050	0.041	0.062	-26.279	0.000
Ahmed et al., 2016	0.027	0.017	0.040	-16.291	0.000
Al-Saeed et al., 2006	0.004	0.002	0.007	-15.898	0.000
Al Shammrie et al., 2017	0.070	0.066	0.074	-77.471	0.000
Alshamrani et al., 2019	0.015	0.010	0.024	-18.021	0.000
Bahamdan et al., 1995	0.032	0.023	0.043	-20.972	0.000
Parthasaradhi et al., 1998	0.041	0.035	0.049	-35.927	0.000
	0.060	0.056	0.063	-95.306	0.000

Event rate and 95% CI

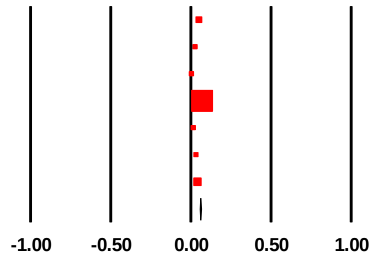

Melasma

Study name	Event rate	Lower limit	Upper limit	Z-Value	p-Value
A. M. Albasri et al., 2019	0.001	0.000	0.006	-7.022	0.000
Alakloby, 2005	0.017	0.012	0.024	-21.383	0.000
Ahmed et al., 2016	0.048	0.035	0.065	-17.971	0.000
Parthasaradhi et al., 1998	0.023	0.018	0.028	-32.195	0.000
	0.025	0.022	0.030	-42.812	0.000

Event rate and 95% CI

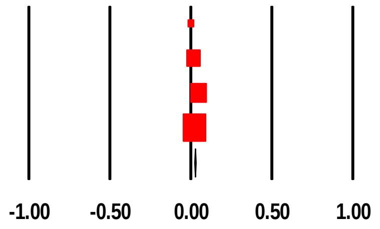

Other Pigmentary Disorders

Study name	Event rate	Lower limit	Upper limit	Z-Value	p-Value
Ahmed et al., 2016	0.003	0.001	0.010	-8.445	0.000
Al-Saeed et al., 2006	0.048	0.040	0.058	-30.235	0.000
Al Shammrie et al., 2017	0.070	0.066	0.074	-77.471	0.000
Alshamrani et al., 2019	0.014	0.009	0.023	-17.779	0.000
Bahamdan et al., 1995	0.016	0.011	0.025	-18.171	0.000
Parthasaradhi et al., 1998	0.007	0.004	0.010	-23.389	0.000
	0.061	0.058	0.064	-89.136	0.000

Event rate and 95% CI

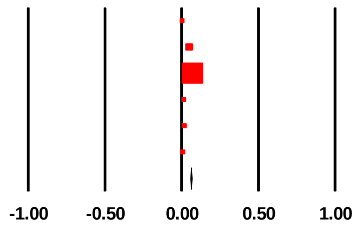

Supplement: Supplementary Materials — Supplementary file 1: risk of bias assessment tool. Supplementary file 2: forest plot of the pooled proportion for the prevalence of pigmentary disorders. Supplementary file 3: forest plot of the pooled proportion for the prevalence of dermatitis/eczema and related conditions. Supplementary file 4: forest plot of the pooled proportion for the prevalence of skin infections. Supplementary file 5: forest plot of the pooled proportion for the prevalence of diseases of skin appendages. Supplementary file 6: forest plot of the pooled proportion for the prevalence of papulosquamous disorders. [file 5281957.f1.zip › 5281957/Supplementary file 2.pdf]
