# Supplementary figures and images for: Epidemiological Patterns of Skin Disease in Saudi Arabia: A Systematic Review and Meta-Analysis
Source: Dermatol Res Pract. 2020 Oct 27;2020:5281957. doi: 10.1155/2020/5281957 (PMC7641721; doi:10.1155/2020/5281957)

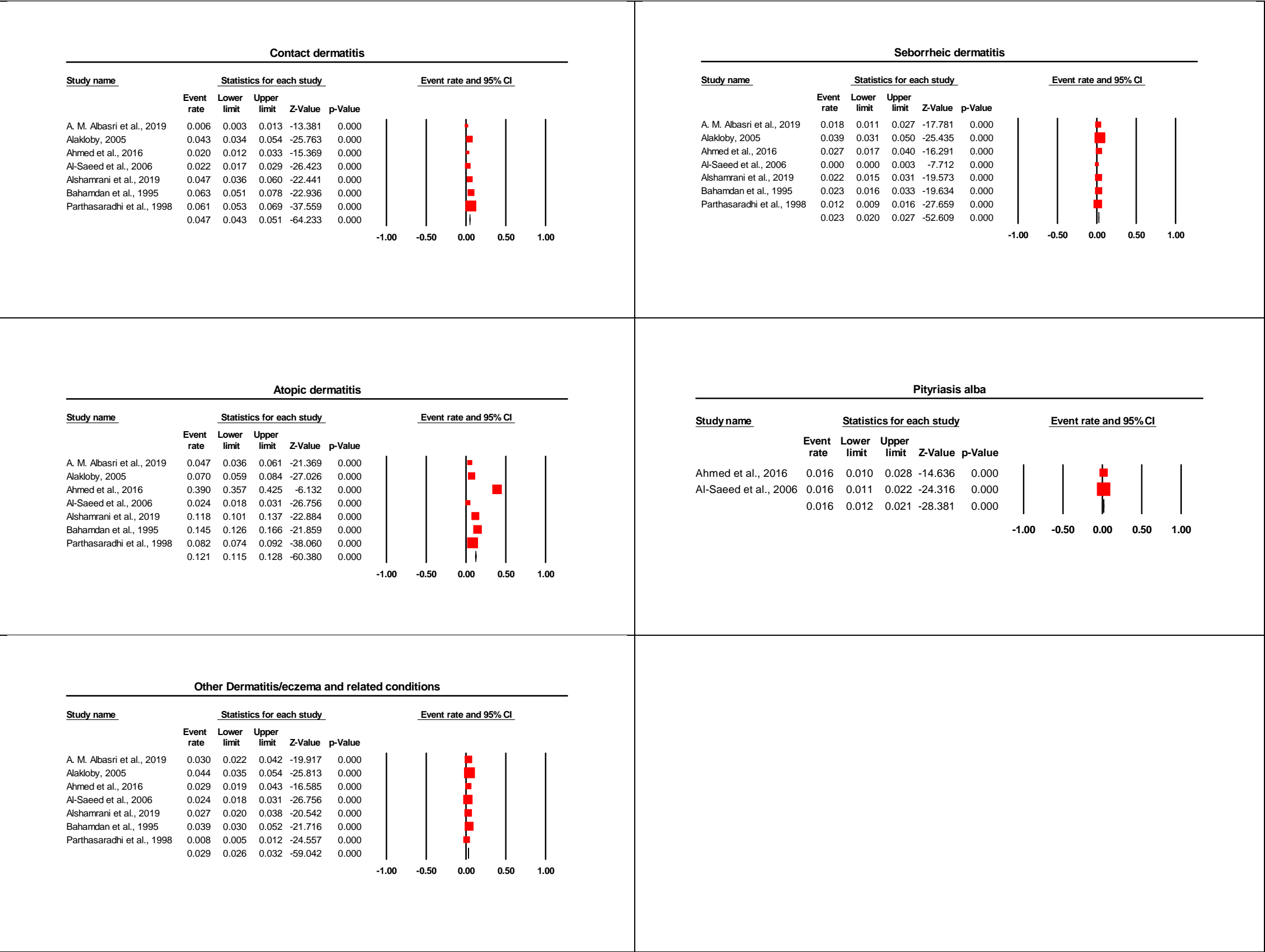

Supplement: Supplementary Materials — Supplementary file 1: risk of bias assessment tool. Supplementary file 2: forest plot of the pooled proportion for the prevalence of pigmentary disorders. Supplementary file 3: forest plot of the pooled proportion for the prevalence of dermatitis/eczema and related conditions. Supplementary file 4: forest plot of the pooled proportion for the prevalence of skin infections. Supplementary file 5: forest plot of the pooled proportion for the prevalence of diseases of skin appendages. Supplementary file 6: forest plot of the pooled proportion for the prevalence of papulosquamous disorders. [file 5281957.f1.zip › 5281957/Supplementary file 3.pdf]

Supplementary File 4

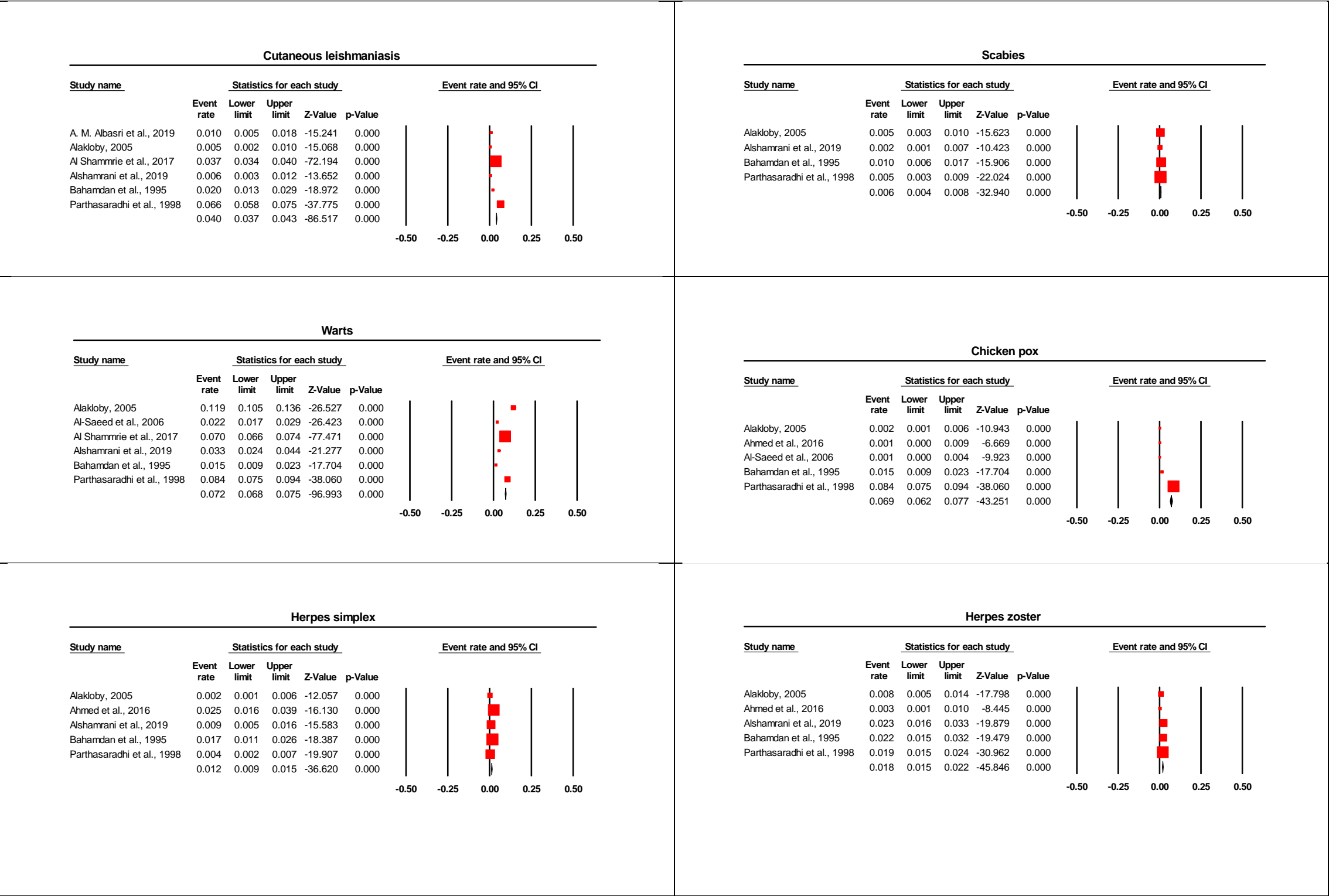

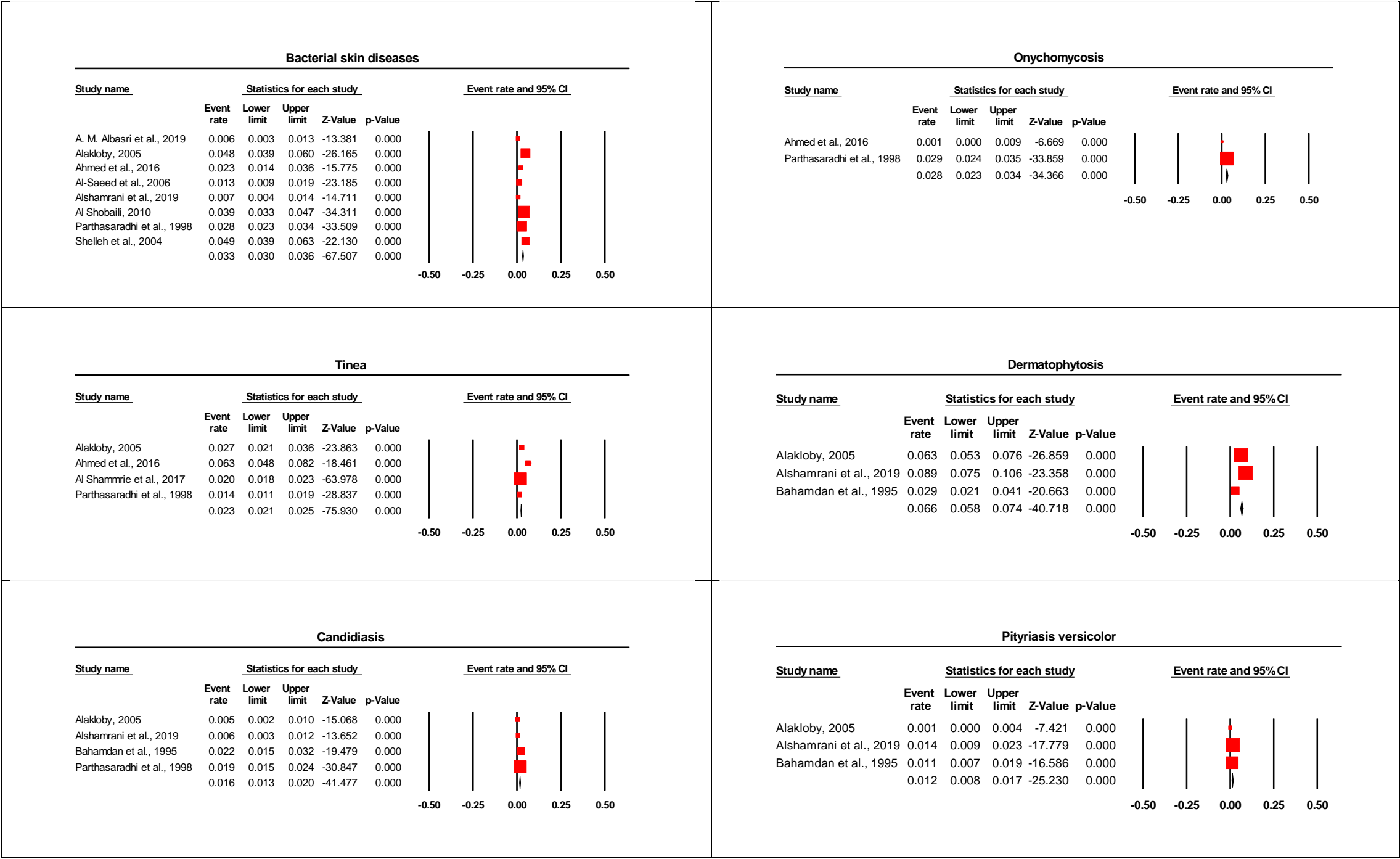

Supplement: Supplementary Materials — Supplementary file 1: risk of bias assessment tool. Supplementary file 2: forest plot of the pooled proportion for the prevalence of pigmentary disorders. Supplementary file 3: forest plot of the pooled proportion for the prevalence of dermatitis/eczema and related conditions. Supplementary file 4: forest plot of the pooled proportion for the prevalence of skin infections. Supplementary file 5: forest plot of the pooled proportion for the prevalence of diseases of skin appendages. Supplementary file 6: forest plot of the pooled proportion for the prevalence of papulosquamous disorders. [file 5281957.f1.zip › 5281957/Supplementary file 4.pdf]

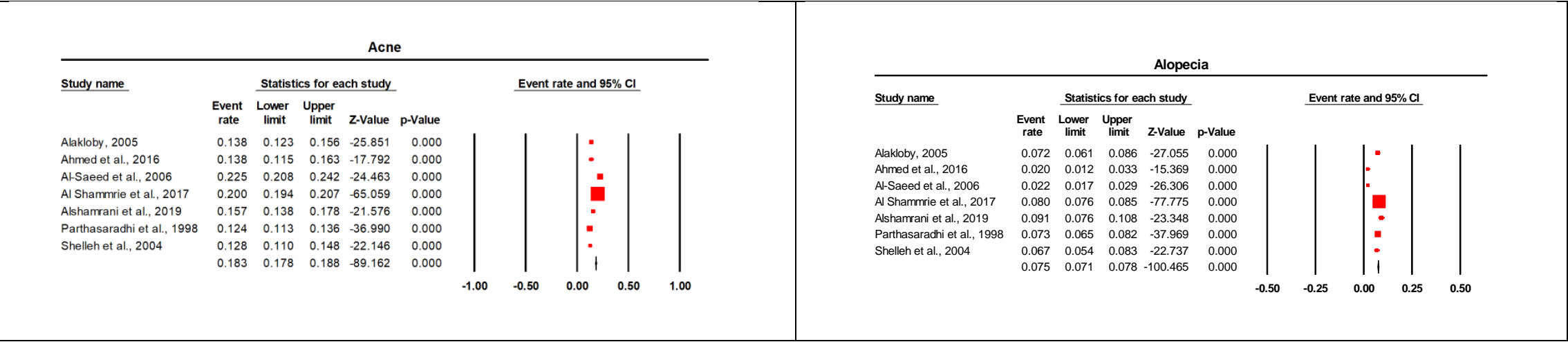

Supplement: Supplementary Materials — Supplementary file 1: risk of bias assessment tool. Supplementary file 2: forest plot of the pooled proportion for the prevalence of pigmentary disorders. Supplementary file 3: forest plot of the pooled proportion for the prevalence of dermatitis/eczema and related conditions. Supplementary file 4: forest plot of the pooled proportion for the prevalence of skin infections. Supplementary file 5: forest plot of the pooled proportion for the prevalence of diseases of skin appendages. Supplementary file 6: forest plot of the pooled proportion for the prevalence of papulosquamous disorders. [file 5281957.f1.zip › 5281957/Supplementary file 5.pdf]

Supplementary File 6

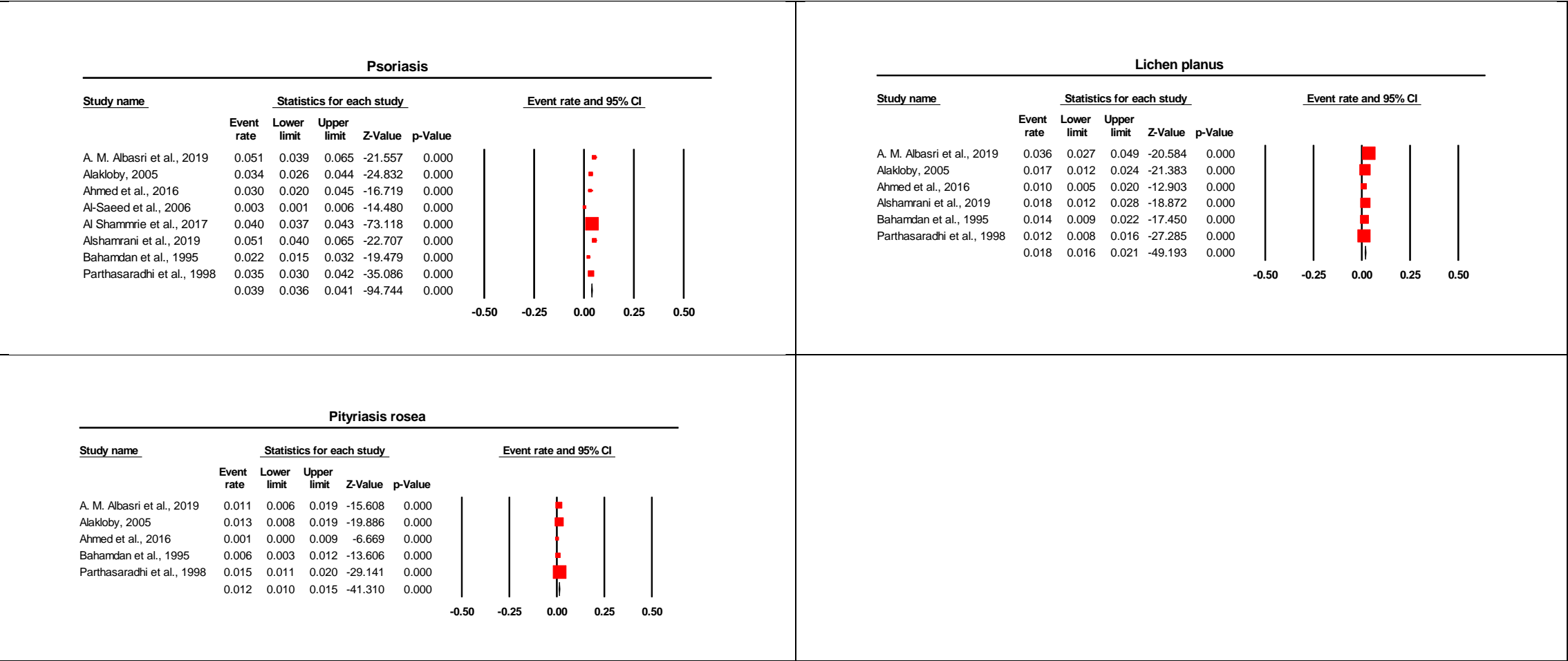

Supplement: Supplementary Materials — Supplementary file 1: risk of bias assessment tool. Supplementary file 2: forest plot of the pooled proportion for the prevalence of pigmentary disorders. Supplementary file 3: forest plot of the pooled proportion for the prevalence of dermatitis/eczema and related conditions. Supplementary file 4: forest plot of the pooled proportion for the prevalence of skin infections. Supplementary file 5: forest plot of the pooled proportion for the prevalence of diseases of skin appendages. Supplementary file 6: forest plot of the pooled proportion for the prevalence of papulosquamous disorders. [file 5281957.f1.zip › 5281957/Supplementary file 6.pdf]
